# Microcrystalline Luminescent (Eu_1-x_Ln_x_)_2_bdc_3_·nH_2_O (Ln = La, Gd, Lu) Antenna MOFs: Effect of Dopant Content on Structure, Particle Morphology, and Luminescent Properties

**DOI:** 10.3390/molecules29020532

**Published:** 2024-01-21

**Authors:** Stefaniia S. Kolesnik, Nikita A. Bogachev, Ilya E. Kolesnikov, Sergey N. Orlov, Mikhail N. Ryazantsev, Gema González, Mikhail Yu. Skripkin, Andrey S. Mereshchenko

**Affiliations:** 1 Saint-Petersburg State University, 7/9 Universitetskaya Emb., 199034 St. Petersburg, Russia; staphylinuscaesareus@gmail.com (S.S.K.); n.bogachev@spbu.ru (N.A.B.); ilya.kolesnikov@spbu.ru (I.E.K.); orlov.s.n.1989@yandex.ru (S.N.O.); mikhail.n.ryazantsev@gmail.com (M.N.R.); skripkin1965@yandex.ru (M.Y.S.); 2 Institute of Nuclear Industry, Peter the Great St. Petersburg Polytechnic University (SPbSU), 29 Polytechnicheskaya Street, 195251 St. Petersburg, Russia; 3 Nanotechnology Research and Education Centre RAS, Saint Petersburg Academic University, ul. Khlopina 8/3, 194021 St. Petersburg, Russia; 4 School of Physical Sciences and Nanotechnology, Yachay Tech University, Urcuqui 100119, Ecuador; ggonzalez@yachaytech.edu.ec

**Keywords:** metal–organic framework, luminescence, microcrystals, rare earth, europium, lutetium, gadolinium, lanthanum, ultrasound

## Abstract

In this work, three series of micro-sized heterometallic europium-containing terephthalate MOFs, (Eu_1-x_Ln_x_)_2_bdc_3_·nH_2_O (Ln = La, Gd, Lu), are synthesized via an ultrasound-assisted method in an aqueous medium. La^3+^ and Gd^3+^-doped terephthalates are isostructural to Eu_2_bdc_3_·4H_2_O. Lu^3+^-doped compounds are isostructural to Eu_2_bdc_3_·4H_2_O with Lu contents lower than 95 at.%. The compounds that are isostructural to Lu_2_bdc_3_·2.5H_2_O are formed at higher Lu^3+^ concentrations for the (Eu_1-x_Lu_x_)_2_bdc_3_·nH_2_O series. All materials consist of micrometer-sized particles. The particle shape is determined by the crystalline phase. All the synthesized samples demonstrate an “antenna” effect: a bright-red emission corresponding to the ^5^D_0_-^7^F_J_ transitions of Eu^3+^ ions is observed upon 310 nm excitation into the singlet electronic excited state of terephthalate ions. The fine structure of the emission spectra is determined by the crystalline phase due to the different local symmetries of the Eu^3+^ ions in the different kinds of crystalline structures. The photoluminescence quantum yield and ^5^D_0_ excited state lifetime of Eu^3+^ are equal to 11 ± 2% and 0.44 ± 0.01 ms, respectively, for the Ln_2_bdc_3_·4H_2_O structures. For the (Eu_1-x_Lu_x_)_2_bdc_3_·2.5H_2_O compounds, significant increases in the photoluminescence quantum yield and ^5^D_0_ excited state lifetime of Eu^3+^ are observed, reaching 23% and 1.62 ms, respectively.

## 1. Introduction

Light-emitting materials based on the metal–organic frameworks (MOFs) of rare earth element (REE) ions have been widely discussed in recent research works because of the wide area of applications, ranging from LEDs and different sensors to bioimaging materials and medicine [1,2,3,4]. For practical application, the particle size of such compounds is one of the most important factors. Small-sized particles (nano- and micro-sized) have a large specific surface area, which provides high sorption properties to compounds and allows them to be used as sensor materials for heavy metal ion detection [3,5,6,7,8]. Such highly dispersed MOFs can be obtained via several synthetic approaches, namely coprecipitation [9], hydrothermal [10], solvothermal [11], sol–gel [12], magnetic-field-induced [13], and some other synthetic methods. One of the simplest, most efficient, and low-cost approaches for the synthesis of small-sized MOF particles is the ultrasound-assisted method, which was recently used to obtain small-sized, REE-based, MOF Eu_2_bdc_3_·4H_2_O nanoparticles, with average particle sizes of 8 ± 2 nm [14]. These nano-sized particles were shown to be one of the most sensitive luminescent MOF-based sensors for Cu^2+^, Cr^3+^, and Fe^3+^ ions. This method of synthesis allows for the preparation of microparticles with certain morphologies and sizes. The ultrasound-assisted method is quite unpopular today but has big prospects because of its advantages in simplicity of use and the ability to control the physical parameters of the target product.

Since the probability of f-f transitions is very small (Laporte’s rule), direct excitation into the 4f excited levels of ions is unfavorable, which limits the application of such luminescent materials. One of the useful approaches to overcome this problem is the so-called “antenna effect”, which suggests using light-harvesting ligands that transfer absorbed energy onto lanthanide ions [15,16]. Typical ligands used in such antenna complexes are aromatic and unsaturated molecules, like calixarenes, bipyridines, phenanthroline derivatives, and carboxylates, including terephthalates [12,17,18,19,20].

The mutual presence of both luminescent and non-luminescent rare earth element (REE) ions can significantly affect the photophysical properties of these compounds. In the mixed terephthalates and diphenylmethionates Eu-Y, Eu-Gd, Tb-Y, and Tb-Gd, it has been shown that the quantum yield and luminescence lifetime significantly depend on the concentration of the non-luminescent ions yttrium and gadolinium [12,17,18], but the reasons that determine the observed effects have practically not been discussed. The number of works reporting the effect of MOF co-doping using non-luminescent REEs is limited, and these works are non-systematic. For example, we have not found any works that have considered the effect of co-doping with lutetium and lanthanum ions (they do not exhibit luminescence) in contrast to gadolinium, which is commonly used as a dopant. In addition, in the works devoted to the study of heterometallic REE-based MOFs containing both luminescent and non-luminescent ions, the mechanism of the mutual effect of REE on the photophysical properties is practically not disclosed. Recently, we reported on heterometallic europium–lutetium and terbium–lutetium terephthalate metal–organic frameworks (MOFs) that demonstrate a strong correlation between the luminescent properties of the complexes and the crystalline structure [19,20]. It has been shown that the luminescence of lanthanide (III) ions strictly depends on the local symmetry of emitting lanthanide ions [19,20,21,22,23,24]. Therefore, in our studies, we have found that the fine structure of luminescence spectra, the lifetimes of excited states, and quantum yields change with crystalline phase change upon varying REE ion contents. For example, in a series of (Tb_x_Lu_1-x_)_2_bdc_3_·nH_2_O compounds (bdc—1,4-benzenedicarboxylate) containing more than 30 at.% of Tb^3+^, only one crystalline phase is formed, Ln_2_bdc_3_·4H_2_O. At lower Tb^3+^ concentrations, terephthalates crystallize as a mixture of Ln_2_bdc_3_·4H_2_O and Ln_2_bdc_3_·10H_2_O or Ln_2_bdc_3_; the exact composition of the product depends on the reagent concentrations used in the synthesis. The ^5^D_4_ excited state Tb^3+^ lifetimes and photoluminescence quantum yield (PLQY) of the anhydrous phase are significantly larger than the corresponding values for terephthalate tetrahydrates and decahydrates. In the case of europium-based compounds, we have found similar results: the luminescence quantum yield of Eu^3+^ was significantly larger for Eu-Lu terephthalates containing a low concentration of Eu^3+^ due to the absence of Eu-Eu energy transfer and the presence of the anhydrous Ln_2_bdc_3_ crystalline phase, with a significantly smaller nonradiative decay rate compared to the Ln_2_bdc_3_·4H_2_O. Therefore, we concluded that the luminescence of Eu^3+^ and Tb^3+^ is quenched by the coordinated water molecules.

To continue our study of the effect of non-luminescent heavy atoms on the interrelated structural and luminescent properties of heterometallic lanthanide-based terephthalates, in the current work, we present the results of our study on microcrystalline europium-based terephthalates doped with lutetium(III), lanthanum(III) and gadolinium(III). In our work, we obtained such MOFs using ultrasound-assisted methods. This unique, simple method allowed us not only to obtain small particles but also to achieve a reproducible synthesis of particles with the desired properties and certain morphology and size. Furthermore, using the ultrasound-assisted method, we obtained a previously unknown crystalline compound, namely Lu_2_bdc_3_·2.5H_2_O, and the isostructural heterometallic Eu-Lu terephthalates, (Eu_1-x_Ln_x_)_2_bdc_3_·2.5H_2_O.

## 2. Results and Discussion

### 2.1. Crystalline Structure

**Figure 1:**
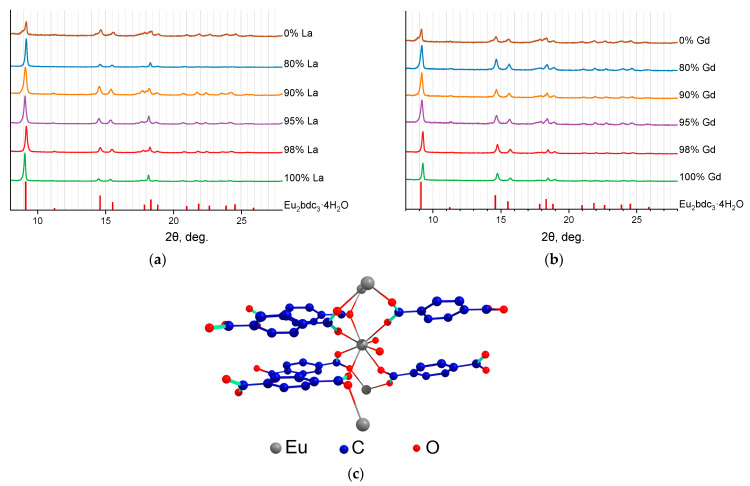
The PXRD patterns of (Eu_1-x_La_x_)_2_bdc_3_·nH_2_O (**a**) and (Eu_1-x_Gd_x_)_2_bdc_3_·nH_2_O (**b**) (x = 0, 0.80, 0.90, 0.95, 0.98, 1) MOFs and the PXRD patterns of Eu_2_bdc_3_·4H_2_O [25] simulated from the single-crystals structures. The La^3+^ and Gd^3+^ contents are shown in the legends. The crystal structure of Eu_2_bdc_3_·4H_2_O adopted from ref. [20] (**c**).

**Figure 2:**
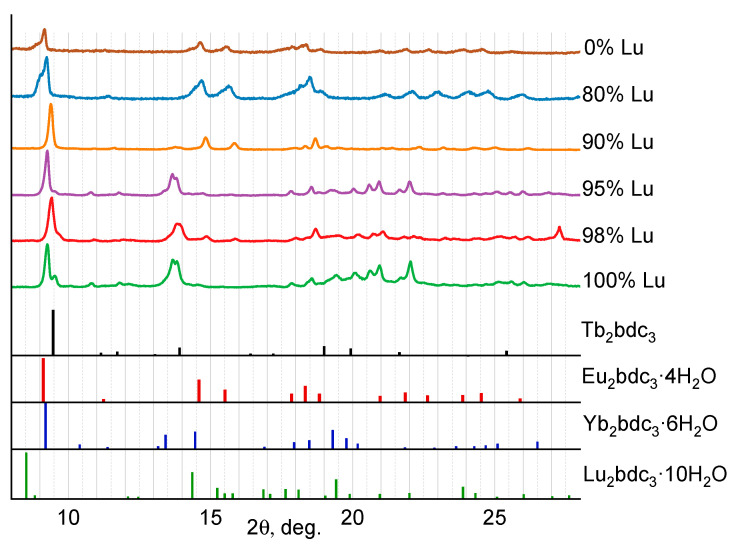
The PXRD patterns of (Eu_1-x_Lu_x_)_2_bdc_3_·nH_2_O (x = 0, 0.80, 0.90, 0.95, 0.98, 1) MOFs and the PXRD patterns of Tb_2_bdc_3_ [25], Eu_2_bdc_3_·4H_2_O [25,26], Yb_2_bdc_3_·6H_2_O [27], Lu_2_bdc_3_·10H_2_O [28] simulated from the single-crystals structures. The Lu^3+^ content is shown in the legend.

In Figure 1a,b, the PXRD patterns of the synthesized MOFs (Eu_1-x_Ln_x_)_2_bdc_3_·nH_2_O (Ln = La, Gd) are shown. We found that mixed Eu-La and Eu-Gd terephthalates were isostructural to the Ln_2_bdc_3_·4H_2_O crystalline phase (Ln = Ce − Yb) [25] at the whole concentration range, and additional peaks were not observed. In the Ln_2_bdc_3_·4H_2_O structure, the lanthanide (III) ions are bound to two water molecules and six terephthalate ions through oxygen atoms, and the Ln^3+^ coordination number (CN) is equal to 8, Figure 1c. The PXRD patterns of (Eu_1-x_Lu_x_)_2_bdc_3_·nH_2_O MOFs are shown in Figure 2. Analysis of the data demonstrates that the structure of the compounds formed depends on the lutetium content. Thus, compounds with a concentration of lutetium (III) ions 90 at.% and less are isostructural to the Ln_2_bdc_3_·4H_2_O crystalline phase. In contrast, when the concentration of lutetium is 95 at.% and more, the positions of the PXRD maxima do not correspond to any known Ln_2_bdc_3_·nH_2_O structure, such as Eu_2_bdc_3_·4H_2_O [25], Tb_2_bdc_3_ [25,26], Yb_2_bdc_3_·6H_2_O [27], Lu_2_bdc_3_·10H_2_O [28], Sc_2_bdc_3_ [29], Er_2_bdc_3_·3H_2_O [30].

**Figure 3:**
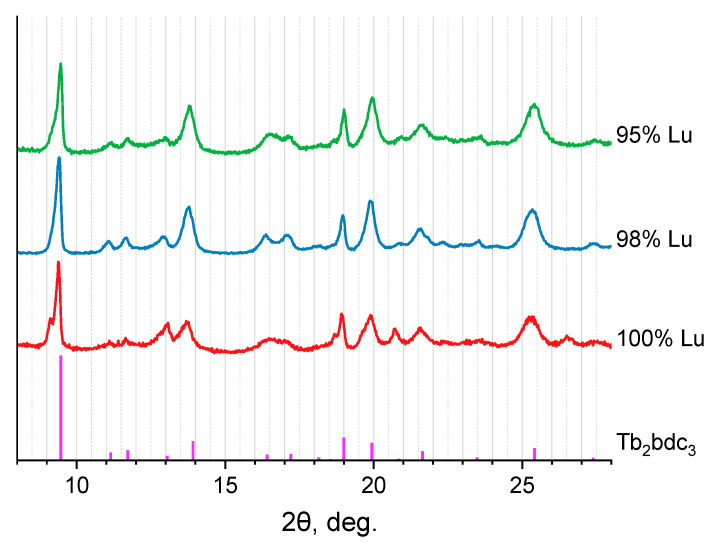
The PXRD patterns of calcinated (Eu_1-x_Lu_x_)_2_bdc_3_·nH_2_O (x = 0.95, 0.98, 1) compounds and the PXRD pattern of Tb_2_bdc_3_ [25] simulated from the single-crystals structure. The Lu^3+^ content is shown in the legend.

**Figure 4:**
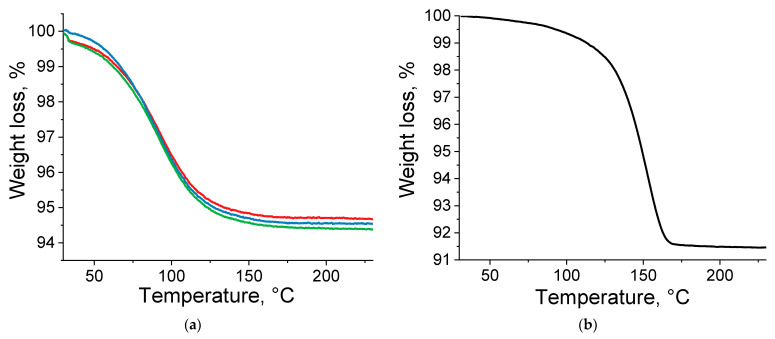
Thermogravimetric curves of Lu_2_bdc_3_·nH_2_O (different line colors correspond to the three parallel measurements) (**a**) and Eu_2_bdc_3_·4H_2_O (**b**).

To prove that compounds containing more than 95 at.% of lutetium have (Eu_1-x_Lu_x_)_2_bdc_3_·nH_2_O composition (containing some water molecules),samples were calcined at the temperature of 200 °C. The positions of the PXRD maxima of the obtained substances correspond to the anhydrous terephthalate structures (Eu_1-x_Lu_x_)_2_bdc_3_, which are isostructural to Tb_2_bdc_3_ (Figure 3). The formation of anhydrous terephthalate is the result of the loss of coordinated water molecules by (Eu_1-x_Lu_x_)_2_bdc_3_·nH_2_O, resulting in the formation of (Eu_1-x_Lu_x_)_2_bdc_3_. Thermogravimetric analysis of Lu_2_bdc_3_·nH_2_O in a temperature range of 30–230 °C allowed us to find the number of coordinated water molecules, n, in the synthesized microcrystalline lutetium terephthalate hydrate Lu_2_bdc_3_·nH_2_O (Figure 4a) under the assumption that this compound was formed in a single crystalline phase. The mass loss was observed at 60–150 °C. As previously reported [19,20], the mass loss in this temperature range can be attributed to the dehydration of the compounds, resulting in the formation of anhydrous terephthalate: Lu_2_bdc_3_·nH_2_O → Lu_2_bdc_3_ + nH_2_O. The average weight loss was found to be (5.31 ± 0.18)%, which corresponds to 2.62 ± 0.09 water molecules per unit formula of Lu_2_bdc_3_·nH_2_O. The product of calcination was assigned to the anhydrous terephthalate based on the PXRD data showing that the calcination product, Lu_2_bdc_3_, is isostructural to Tb_2_bdc_3_, Figure 3. Therefore, we proposed that the new unknown crystalline phase of obtained lutetium terephthalate hydrate has the composition of Lu_2_bdc_3_·2.5H_2_O. This crystalline phase dominates at the lutetium content of 95 at.% and more in our samples. Thermogravimetric analysis of Eu_2_bdc_3_·4H_2_O (Figure 4b) confirmed the number of water molecules per unit formula of europium(III) terephthalate hydrate, where weight loss of 8.51% (5.31% ± 0.18) corresponds to 4.32 water molecules. Most probably, the thermogravimetric analysis gives overestimated values of the number of water molecules per unit formula of metal terephthalate because of the presence of a small amount of the absorbed water in the MOF pores.

Unit cell parameters were refined using UnitCell software [31], Table 1. This program can retrieve unit cell parameters from diffraction data using a least-squares method from the positions of the indexed diffraction maxima of PXRD patterns (Pawley method [32]). For the compounds (Eu_1-x_La_x_)_2_bdc_3_·4H_2_O, the increase in La^3+^ content leads to unit cell volumes increase due to a higher ionic radius of La^3+^ ions (1.160 Å, the coordination number is eight) than the ionic radius of Eu^3+^ ions (1.066 Å) [33]. The ionic radius of the Gd^3+^ ion (1.053 Å) is close to that of Eu^3+^. Therefore, the unit cell parameters do not change significantly in the (Eu_1-x_Gd_x_)_2_bdc_3_·4H_2_O series. The Lu^3+^ ion (ionic radius is 0.977 Å) is smaller than Eu^3+^; therefore, substitution of Eu^3+^ by Lu^3+^ ion results in a decrease in the unit cell volumes in (Eu_1-x_Lu_x_)_2_bdc_3_·4H_2_O series with the lutetium (III) ions content of 90 at.% and less, where the MOF is formed as tetrahydrate, Ln_2_bdc_3_·4H_2_O.

### 2.2. Particle Morphology

**Figure 5:**
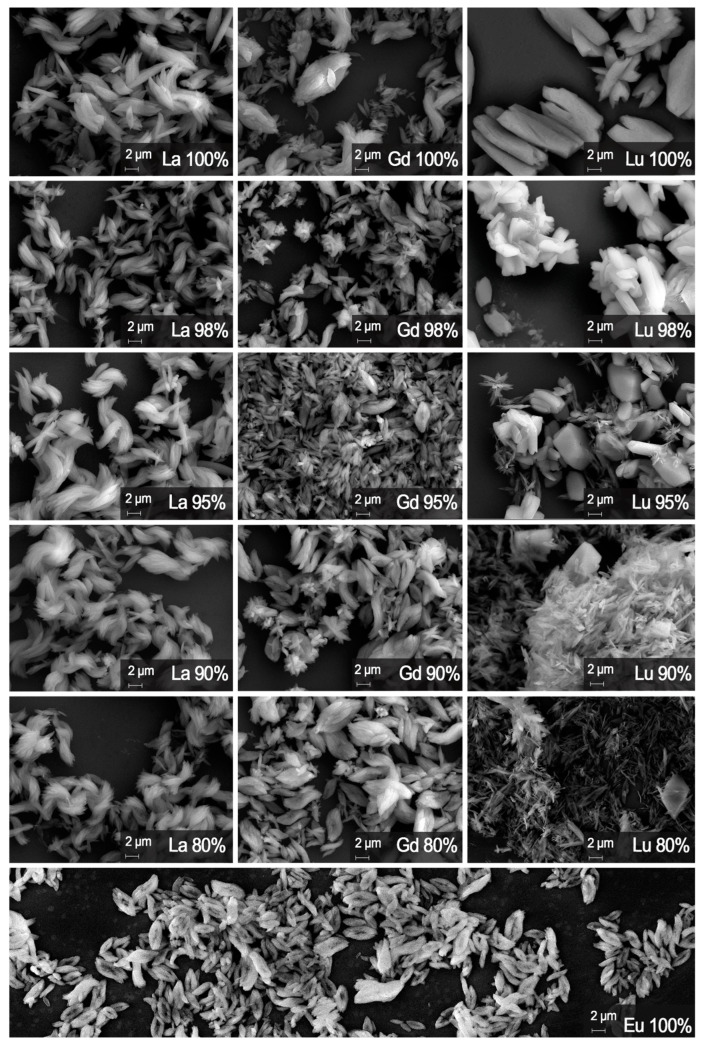
SEM images of the samples (Eu_1-x_Ln_x_)_2_bdc_3_·nH_2_O (Ln = Lu, La, Gd).

Scanning electron microscopy (SEM) was used to observe the shape and the size of the particles of the synthesized MOFs (Eu_1-x_Ln_x_)_2_bdc_3_·nH_2_O (Ln = La, Gd, Lu). The SEM images are shown in Figure 5, where (Eu_1-x_La_x_)_2_bdc_3_·nH_2_O, (Eu_1-x_Gd_x_)_2_bdc_3_·nH_2_O, (Eu_1-x_Lu_x_)_2_bdc_3_·nH_2_O compounds are presented in the left, central, and right columns, respectively. The length and width of the particles obtained from the SEM images are given in Table 2. In the (Eu_1-x_La_x_)_2_bdc_3_·4H_2_O and (Eu_1-x_Gd_x_)_2_bdc_3_·4H_2_O series, the particles have a similar oval plate shape, which can resemble one a petal or a leaf, as well as a similar size of about 6 × 2 μm. We noticed that the particles have different shapes depending on the content of lutetium ion in the (Eu_1-x_Lu_x_)_2_bdc_3_·nH_2_O series. Thus, at the Lu^3+^ content below 90 at.%, the particles have the shape of rods and a size of about 4 × 0.8 μm. However, in (Eu_1-x_Lu_x_)_2_bdc_3_·nH_2_O MOFs with a concentration of Lu^3+^ more than 90%, the particles are “brick”-shaped and are significantly larger, about 10 × 5 μm. At the Lu^3+^ concentration of 90 and 95 at.%, the mixture of “bricks” and “rods” is observed. The shape difference probably results from the change in the crystalline structure at the Lu^3+^ concentration of 95% among the (Eu_1-x_Lu_x_)_2_bdc_3_·nH_2_O series. Detailed analysis of the shape (Eu_1-x_La_x_)_2_bdc_3_·4H_2_O and (Eu_1-x_Gd_x_)_2_bdc_3_·4H_2_O microparticles revealed that leaf-shaped microparticles are made of rods, whereas the separate rods about of the same size are observed in the (Eu_1-x_Lu_x_)_2_bdc_3_·4H_2_O (x = 0.8, 0.9) samples. We propose that the reason might be in different dopant properties. Lu^3+^ is the smallest ion among the trivalent lanthanide cations [33]. Therefore, the Lu^3+^ ion possesses the highest positive charge density among lanthanides. Therefore, we propose that the lutetium terephthalate surface has the highest surface charge density among the series. In the literature [34], a higher surface charge density corresponds to a higher colloidal stability of the solution. We believe that small rod”-shaped particles initially form in the mixed terephthalates containing 80–90 at.% dopant. Therefore, Gd and La-doped particles coagulate, forming “leaf”-shaped particles, whereas Lu-doped particles are still colloidally stable and save their “rod” form if they correspond to the Ln_2_bdc_3_·4H_2_O crystalline phase.

It is worth noting that the ultrasound-assisted method allowed us to obtain microparticles with a very small size variance (near 1–3 μm). This result suggests that using this method, it is possible to obtain particles with a certain morphology and size by changing the dopant content.

### 2.3. Luminescence Properties

Europium and terbium terephthalates demonstrate a pronounced antenna effect where terephthalate ion as a synthesizer or an “antenna” effectively absorbs UV radiation and transfers energy to a luminescent lanthanide ion followed by the metal-centered emission [18]. Upon the excitation, the terephthalate ion is promoted into the S_n_ state, followed by the fast internal conversion to the S_1_ state. Due to the heavy atom effect caused by the lanthanide atom, the S_1_ state efficiently undergoes intersystem crossing to the T_1_ triplet electronic excited state [18]. The T_1_ state of the terephthalate ion is close in energy to the ^5^D_1_ energy level of the Eu^3+^ ion. Therefore, an efficient energy transfer from sensitizer to luminescent lanthanide ion occurs. The ^5^D_1_ level of the Eu^3+^ then undergoes an internal conversion into the ^5^D_0_ state, followed by the emission into the ^7^F_J_ (J = 0–4) lower-lying energy levels.

**Figure 6:**
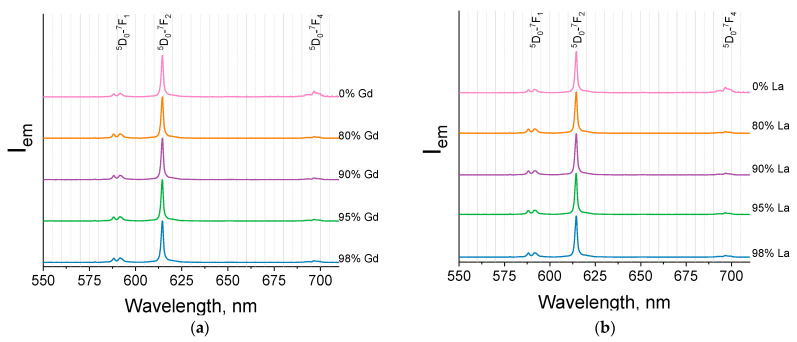
Emission spectra of (Eu_1-x_Gd_x_)_2_bdc_3_·nH_2_O (**a**) and (Eu_1-x_La_x_)_2_bdc_3_·nH_2_O (**b**) (x = 0, 0.80, 0.90, 0.95, 0.98) compounds upon 310-nm excitation. The La^3+^ and Gd^3+^ contents are shown in the legends.

**Figure 7:**
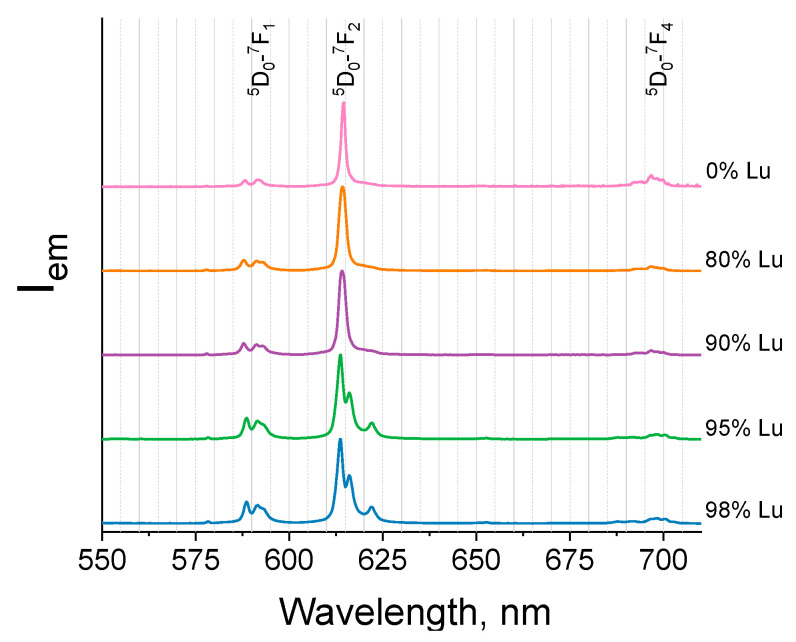
Emission spectra of the (Eu_1-x_Lu_x_)_2_bdc_3_·nH_2_O nH_2_O (x = 0, 0.80, 0.90, 0.95, 0.98) compounds upon 310-nm excitation. The Lu^3+^ content is shown in the legend.

For the synthesized luminescent materials (Eu_1-x_Ln_x_)_2_bdc_3_·nH_2_O (Ln = La, Gd, Lu and x = 0, 0.80, 0.90, 0.95, 0.98), emission spectra were measured upon 310-nm excitation (Figure 6 and Figure 7). The emission spectra for the Gd and La-doped compounds are similar to the pure europium terephthalateemission spectrum. This observation agrees with the PXRD data showing that (Eu_1-x_Ln_x_)_2_bdc_3_·nH_2_O (Ln = La, Gd) compounds are isostructural to Eu_2_bdc_3_·4H_2_O compound (Figure 6). The emission spectra of Gd and La-doped terephthalates consist of the narrow bands corresponding to ^5^D_0_-^7^F_J_ (J = 0–4) transitions of Eu^3+^: ^5^D_0_-^7^F_1_ (588.2 and 591.6 nm), ^5^D_0_-^7^F_2_ (614.6 nm), and ^5^D_0_-^7^F_4_ (696.6 nm). ^5^D_0_-^7^F_0_ and ^5^D_0_-^7^F_3_ bands were not observed in the emission spectra due to their weak intensity.

The emission spectra of the (Eu_1-x_Lu_x_)_2_bdc_3_·nH_2_O compounds are shown in Figure 7. This figure clearly shows the difference in the fine structure of the measured emission spectra for the Lu concentration of 0, 80, 90, 95, and 98 at.%. The first three emission spectra are identical to the spectrum of Eu_2_bdc_3_·4H_2_O. This observation is consistent with PXRD data demonstrating that (Eu_1-x_Lu_x_)_2_bdc_3_·nH_2_O compounds (x = 0.80, 0.90) crystalize in Eu_2_bdc_3_·4H_2_O phase without formation of any additional phase. The emission spectra of the (Eu_1-x_Lu_x_)_2_bdc_3_·nH_2_O compounds consist of narrow bands corresponding to ^5^D_0_-^7^F_1_ (587.9, 591.3, and 591.8 nm), ^5^D_0_-^7^F_2_ (614.2 nm), and ^5^D_0_-^7^F_4_ (696.7 nm). On the contrary, the emission spectra of the (Eu_1-x_Lu_x_)_2_bdc_3_·nH_2_O (x = 0.95, 0.98) compounds are different from the spectrum of pure europium terephthalate. Such difference can be explained by the formation of another phase, namely Ln_2_bdc_3_·2.5H_2_O, where the local symmetry of the europium (III) ion is different from that of Eu_2_bdc_3_·4H_2_O. These spectra consist of narrow bands corresponding to ^5^D_0_-^7^F_1_ (588.6, 591.6, and 593 nm), ^5^D_0_-^7^F_2_ (613.7, 616.0, and 622.0 nm), and ^5^D_0_-^7^F_4_ (698.2 nm).

**Figure 8:**
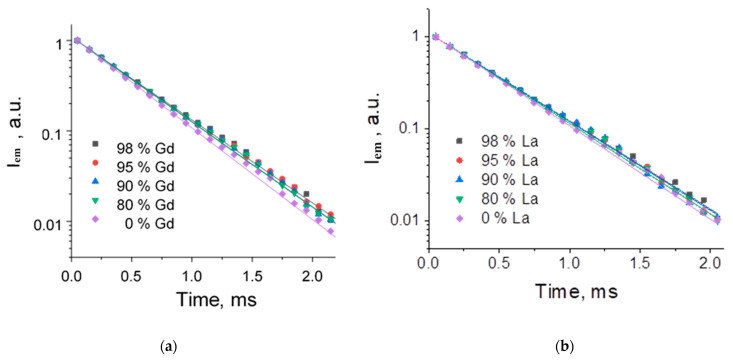
The photoluminescence decay curves of the (Eu_1-x_Gd_x_)_2_bdc_3_·nH_2_O (**a**) and (Eu_1-x_La_x_)_2_bdc_3_·nH_2_O (**b**) (x = 0, 0.80, 0.90, 0.95, 0.98) compounds monitored at 614 nm upon 310-nm excitation.

**Figure 9:**
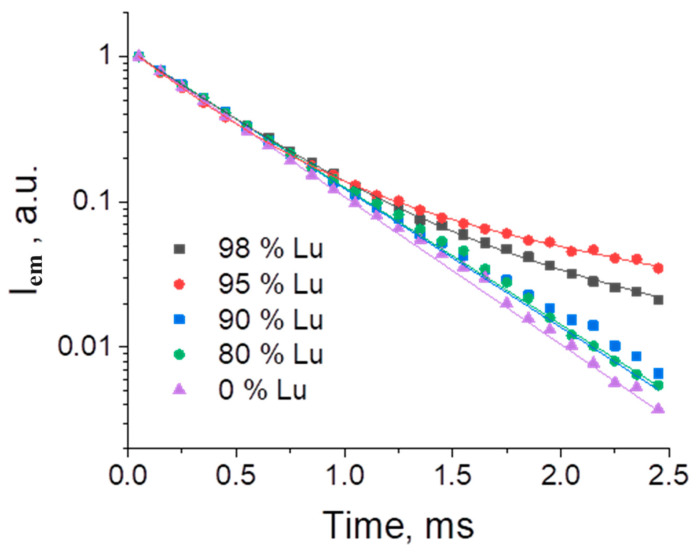
The photoluminescence decay curves of (Eu_1-x_Lu_x_)_2_bdc_3_·nH_2_O nH_2_O (x = 0, 0.80, 0.90, 0.95, 0.98) were monitored at 614 nm.

The photoluminescence decay curves were measured under UV-excitation and monitored at 614 nm (^5^D_0_-^7^F_2_) for the (Eu_1-x_Ln_x_)_2_bdc_3_·nH_2_O (Ln = La, Gd, Lu and x = 0, 0.80, 0.90, 0.95, 0.98) compounds (Figure 8 and Figure 9). The photoluminescence decay curves of La and Gd doped terephthalates (Figure 8) were accurately fitted by single exponential functions (Equation (1)), resulting in time constants *τ* of about 0.43–0.47 ms for the Gd doped terephthalates and about 0.43–0.45 ms for the La-doped terephthalates (Table 3). For the (Eu_1-x_Lu_x_)_2_bdc_3_·nH_2_O materials, the photoluminescence decay curves were fitted by the single exponential functions (Equation (1)) at the lutetium content less than 90 at.% resulting in time constants *τ* of about 0.43–0.46 ms (Figure 9). Meanwhile, at lutetium concentrations more than 90 at.% The photoluminescence decay curves can be fitted only using the double exponential functions (Equation (2)) with the time constants *τ*_1_ of about 0.34–0.46 ms and *τ*_2_ of about 1.5 ms. The two-exponential decay of the highly doped Eu-Lu terephthalates (Eu_1-x_Lu_x_)_2_bdc_3_·nH_2_O (x = 0.95, 0.98) indicates the two different coordination sites of the Eu^3+^ ion. The fast component (0.3–0.5 ms) is close to that of the water-coordinated Eu^3+^ ion in the Ln_2_bdc_3_·4H_2_O structure, whereas the slow component (1.5–1.6 ms) corresponds to the Eu^3+^ ion exclusively coordinated to carboxylic groups of terephthalate ions [20] due to the absence of luminescence quenching of Eu^3+^ by coordinated water molecules containing high-frequency OH vibrational modes.

For all synthesized materials, photoluminescence quantum yields (PLQYs) were measured (Table 3). PLQYs for the Gd and La-doped terephthalates are found to be approximately equal. But for the (Eu_1-x_Lu_x_)_2_bdc_3_·nH_2_O materials, PLQYs grow with the Lu concentration increase and reach a maximum of 23% at the 95 at.% Lu. The significantly higher values of photoluminescence quantum yield in Eu-Lu terephthalates at high Lu content can be explained by the presence in these materials’ Eu^3+^ coordination sites bound only to carboxylic groups of terephthalate ions [20] and not to the water molecules, which quenches the Eu^3+^ luminescence.

(1) $$I=I_{1}\cdot e^{-\frac{t}{\tau }}$$

(2) $$I=I_{1}\cdot e^{-\frac{t}{\tau_{1}}}+I_{2}\cdot e^{-\frac{t}{\tau_{2}}}$$

Due to the different nature of the f-f transitions of Eu^3+^ ions, these ions can be used as structural probes [35,36]. Thus, the probability of hypersensitive ^5^D_0_-^7^F_2_ forced electric dipole transition is strongly affected by the local environment of the europium ion, whereas the probability of ^5^D_0_-^7^F_1_ magnetic dipole transition intensity is significantly less sensitive to changes in the Eu^3+^ coordination sphere. The analysis of asymmetry ratio R_as_, which is equal to the ratio of the integral intensity of these transitions (^5^D_0_-^7^F_2_)/(^5^D_0_-^7^F_1_), allows one to track the changes in the local environment of the europium (III) ions [37,38]. The higher values of the asymmetry ratio correspond to the greater deviation of the Eu^3+^ environment from the centrosymmetric one [39]. The asymmetry ratio for the synthesized compounds is given in Table 4. The asymmetry ratio decreases with the increasing dopant concentration caused by the distortion of the crystal structure near the luminescent centers. The lutetium-doped materials have a minimal asymmetry ratio at the whole concentration range of the dopant, especially when the concentration of lutetium is 95 at.% and even more when the phase transition occurs. The reason for this effect is that the lutetium (III) ion has a smaller ionic radius than the gadolinium(III) and lanthanum(III) doping ions, and the environment of the luminescent center is not distorted. Lanthanum and gadolinium-doped terephthalates have nearly equal asymmetry ratios because of the almost identical structures of these materials.

## 3. Materials and Methods

### 3.1. Materials

Lutetium (III) chloride hexahydrate (>99%), gadolinium chloride hexahydrate (>99%), lanthanum chloride hexahydrate (>99%), and europium (III) chloride hexahydrate (>99%) were purchased from Chemcraft (Kaliningrad, Russia). Benzene-1,4-dicarboxylic (terephthalic, H_2_bdc) acid (>98%), sodium hydroxide (>99%), nickel (II) chloride hexahydrate (>99%), and EDTA disodium salt (0.1 M aqueous solution) were purchased from Sigma-Aldrich Pty Ltd. (Taufkirchen, Germany) and used without additional purification. The 0.3 M aqueous solution of disodium terephthalate (Na_2_bdc) was prepared by dissolving terephthalic acid in an aqueous sodium hydroxide solution and then diluted to obtain the 10 mM Na_2_bdc aqueous solution. The 0.2 M EuCl_3_ LuCl_3_, LaCl_3_, and GdCl_3_ solutions were prepared, standardized using back complexometric titration as described earlier [40], and then diluted to obtain the solutions containing the mixture of REE chlorides used in the synthesis of MOFs (Table 5).

### 3.2. Synthesis

In the current work, three series of microcrystalline heterometallic MOFs were synthesized, namely (Eu_1-x_La_x_)_2_bdc_3_·nH_2_O, (Eu_1-x_Gd_x_)_2_bdc_3_·nH_2_O, and (Eu_1-x_Lu_x_)_2_bdc_3_·nH_2_O (x = 0, 0.80, 0.90, 0.95, 0.98, 1). The MOFs were synthesized by a slow dropwise addition of 10 mL of 10 mM Na_2_bdc aqueous solution to the 10 mL solution containing chlorides of the abovementioned lanthanides with a total REE concentration of 5 mM accompanied by ultrasonication (40 kHz, 60 W) and vigorous stirring. The initial concentrations of REE ions are given in Table 5. After the dropwise addition that took 5 min, the reaction mixture was kept in an ultrasonic bath for 10 more minutes. As a result, white precipitates of europium(III)-lanthanide(III) terephthalates were formed. The MOFs were separated from the reaction mixture by centrifugation (4000 g) and washed with distilled water five times. The resulting materials were dried under reduced pressure (0.02 atm.) at 20 °C in the desiccator for 1 day. As a result, heterometallic MOFs (Eu_1-x_Ln_x_)_2_bdc_3_·nH_2_O (Ln = Lu, La, Gd and x = 0, 0.80, 0.90, 0.95, 0.98, 1) were obtained. Ratios of the rare earth elements in the synthesized compounds were confirmed by the EDX method (Table 6). EDX shows that, in general, the ratio of elements taken for synthesis is maintained during crystallization.

### 3.3. Methods

The relative content of the rare earth elements in the synthesized compounds was confirmed by energy-dispersive X-ray spectroscopy. The morphology of the particles was characterized by scanning electron microscopy (SEM) on a Zeiss Merlin electron microscope (Zeiss, Oberkochen, Germany) using an energy-dispersive X-ray spectroscopy (EDX) module (Oxford Instruments INCAx-act, High Wycombe, UK). Powder X-ray diffraction (PXRD) measurements were performed on a D2 Phaser (Bruker, Billerica, MA, USA) X-ray diffractometer using Cu Ka radiation (λ = 1.54056 Å). The thermal behavior of the compounds was studied by means of thermogravimetry using a Thermo-microbalance TG 209 F1 Libra (Netzsch, Selb, Germany) with a heat-up rate of 10 °C/min. To carry out photoluminescence studies, the synthesized samples (20 mg) and potassium bromide (300 mg) were pressed into pellets (diameter 13 mm). The luminescence spectra were recorded on Fluorolog-3 fluorescence spectrometer (Horiba Jobin Yvon, Kyoto, Japan). Lifetime measurements were performed using the same spectrometer using a pulsed Xe lamp (pulse duration 3 µs).

## 4. Conclusions

In this article, we reported on the morphology and the photoluminescence properties of the three series of microcrystalline heterometallic europium-containing terephthalate metal–organic frameworks synthesized by ultrasound-assisted method from diluted aqueous solutions: (Eu_1-x_La_x_)_2_bdc_3_·nH_2_O, (Eu_1-x_Gd_x_)_2_bdc_3_·nH_2_O and (Eu_1-x_Lu_x_)_2_bdc_3_·nH_2_O (x = 0–1). The effect of the dopant concentration on the structural properties was revealed. Thus, the La^3+^ and Gd^3+^-doped terephthalates are isostructural to Eu_2_bdc_3_·4H_2_O, but the Lu^3+^ doped compounds are isostructural to Eu_2_bdc_3_·4H_2_O only when the Lu content is lower than 95 at.%; at higher Lu^3+^ content, the new structure namely Lu_2_bdc_3_·2.5H_2_O was obtained. The unit cell parameters were optimized, and the unit cell parameters were refined for the compounds corresponding to Ln_2_bdc_3_·4H_2_O crystalline phase. Unit cell parameters strongly depend on the content and ionic radius of the doping ion in the (Eu_1-x_La_x_)_2_bdc_3_·nH_2_O and (Eu_1-x_Lu_x_)_2_bdc_3_·nH_2_O series. Thus, the substitution of Eu^3+^ for larger La^3+^ ions increases the unit cell volumes, whereas doping by the smaller Lu^3+^ ion results in a decrease in the unit cell volume. This effect is more pronounced for a higher concentration of doping ions. The ionic radius of the Gd^3+^ ion is close to that of Eu^3+^. Therefore, the unit cell parameters do not change significantly in the (Eu_1-x_Gd_x_)_2_bdc_3_·4H_2_O series. The analysis of the morphology of the synthesized materials by scanning electron microscopy has demonstrated that the ultrasound-assisted method results in the formation of particles that have a size of several micrometers and a shape determined by the crystalline phase. In the (Eu_1-x_La_x_)_2_bdc_3_·4H_2_O and (Eu_1-x_Gd_x_)_2_bdc_3_·4H_2_O series, the particles have a similar “leaf” shape and size of approximately 6 × 2 μm. In the (Eu_1-x_Lu_x_)_2_bdc_3_·nH_2_O series, particles have different shapes depending on the content of lutetium ion. At the Lu^3+^ content below 90 at.%, which corresponds to the Ln_2_bdc_3_·4H_2_O crystalline phase, the particles have the shape of rods and a size of approximately 4 × 0.8 μm. However, in (Eu_1-x_Lu_x_)_2_bdc_3_·nH_2_O MOFs with a concentration of Lu^3+^ more than 90% (Ln_2_bdc_3_·2.5H_2_O crystalline phase), the particles are “brick”-shaped and significantly larger, about 10 × 5 μm. At the Lu^3+^ concentration of 90 and 95 at.%, the mixture of “bricks” and “rods” is observed.

All synthesized samples containing Eu^3+^ demonstrated a bright-red emission corresponding to the ^5^D_0_-^7^F_J_ (J = 1, 2, 4) transitions of Eu^3+^ ions upon 310-nm excitation to the singlet electronic excited state of terephthalate ions due to the “antenna” effect. The fine structure of the emission spectra is determined by the crystalline phase due to the different local symmetry of the Eu^3+^ ions in different types of crystalline structure, namely Ln_2_bdc_3_·4H_2_O and Ln_2_bdc_3_·2.5H_2_O. In the (Eu_1-x_La_x_)_2_bdc_3_·nH_2_O and (Eu_1-x_Gd_x_)_2_bdc_3_·nH_2_O series, the photoluminescence quantum yields and ^5^D_0_ excited state lifetimes are equal to 11 ± 2% and 0.44 ± 0.01 ms, respectively, and almost do not depend on the content of La^3+^ and Gd^3+^. The substitution of Eu^3+^ for Lu^3+^ results in an increase in both the photoluminescence quantum yield (up to 23% at the Lu^3+^ content of 95%) and ^5^D_0_ excited state lifetime (up to 1.62 ms) in agreement with the phase transition from Ln_2_bdc_3_·4H_2_O to Ln_2_bdc_3_·2.5H_2_O. Therefore, in the current work, we have shown that the ultrasound-assisted method allows us to control the morphology of the particles and the luminescent properties of selected heterometallic antenna MOFs (Eu_1-x_Ln_x_)_2_bdc_3_·nH_2_O (Ln = La, Gd, Lu) by changing the concentration of doping REE ion. This outstanding result will allow one to obtain particles with predetermined physical properties, morphology, and size. Furthermore, using the ultrasound-assisted method, we discovered the new previously unknown crystalline compound, namely Lu_2_bdc_3_·2.5H_2_O, and the isostructural heterometallic Eu-Lu terephthalates, (Eu_1-x_Ln_x_)_2_bdc_3_·2.5H_2_O, which can be synthesized exclusively by our synthetic procedure under ultrasonic conditions.

## Figures and Tables

**Table 1:** Unit cell parameters with calculation errors for (Eu_1-x_Ln_x_)_2_bdc_3_·nH_2_O (Ln = Lu, La, Gd) refined for Tb_2_bdc_3_·4H_2_O crystalline phase.

χ_Lu_ (at.%)	a, Å	b, Å	c, Å	α	β	γ	V, Å^3^
0	6.1860 ± 0.0018	10.103 ± 0.003	10.184 ± 0.003	102.279 ± 0.026	91.423 ± 0.027	101.482 ± 0.028	608.00 ± 0.24
80	6.1435 ± 0.0018	10.014 ± 0.003	10.061 ± 0.003	101.985 ± 0.026	91.683 ± 0.026	101.232 ± 0.028	592.26 ± 0.23
90	6.0896 ± 0.0018	9.994 ± 0.003	9.971 ± 0.003	101.466 ± 0.026	91.631 ± 0.026	100.876 ± 0.028	582.66 ± 0.23
**χ_La_ (at.%)**	**a, Å**	**b, Å**	**c, Å**	**α**	**β**	**γ**	**V, Å^3^**
0	6.1860 ± 0.0018	10.103 ± 0.003	10.184 ± 0.003	102.279 ± 0.026	91.423 ± 0.027	101.482 ± 0.028	608.00 ± 0.24
80	6.2261 ± 0.0018	10.142 ± 0.003	10.273 ± 0.003	102.127 ± 0.026	91.657 ± 0.027	101.507 ± 0.028	619.84 ± 0.24
90	6.2477 ± 0.0019	10.198 ± 0.004	10.292 ± 0.003	102.169 ± 0.026	91.599 ± 0.027	101.513 ± 0.028	626.41 ± 0.25
95	6.2632 ± 0.0019	10.197 ± 0.004	10.308 ± 0.003	102.235 ± 0.026	91.537 ± 0.027	101.565 ± 0.028	628.68 ± 0.25
98	6.2412 ± 0.0005	10.159 ± 0.008	10.283 ± 0.013	102.32± 0.18	91.53 ± 0.15	101.78 ± 0.12	621.85 ± 0.25
100	6.2691 ± 0.0019	10.206 ± 0.004	10.336 ± 0.003	102.260 ± 0.026	91.552 ± 0.027	101.495 ± 0.028	631.61 ± 0.25
**χ_Gd_ (at.%)**	**a, Å**	**b, Å**	**c, Å**	**α**	**β**	**γ**	**V, Å^3^**
0	6.1860 ± 0.0018	10.103 ± 0.003	10.184 ± 0.003	102.279 ± 0.026	91.423 ± 0.027	101.482 ± 0.028	608.00 ± 0.24
80	6.2368 ± 0.0019	10.036 ± 0.003	10.251 ± 0.003	102.879 ± 0.026	91.989 ± 0.027	101.617 ± 0.028	610.54 ± 0.23
90	6.1611 ± 0.0018	10.077 ± 0.003	10.148 ± 0.003	102.212 ± 0.026	91.258 ± 0.026	101.411 ± 0.028	602.25 ± 0.23
95	6.2354 ± 0.0019	10.031 ± 0.003	10.234 ± 0.003	102.829 ± 0.026	91.931 ± 0.027	101.480 ± 0.028	609.61 ± 0.23
98	6.2235 ± 0.0018	9.995 ± 0.003	10.238 ± 0.003	103.065 ± 0.026	91.812 ± 0.027	101.641 ± 0.027	605.60 ± 0.23
100	6.2112 ± 0.0018	9.992 ± 0.003	10.227 ± 0.003	102.810 ± 0.026	92.048 ± 0.027	101.481 ± 0.027	604.40 ± 0.23

**Table 2:** Sizes of particles of the samples (Eu_1-x_La_x_)_2_bdc_3_·nH_2_O, (Eu_1-x_Gd_x_)_2_bdc_3_·nH_2_O, and (Eu_1-x_Lu_x_)_2_bdc_3_·nH_2_O.

(Eu_1-x_La_x_)_2_bdc_3_·nH_2_O			(Eu_1-x_Gd_x_)_2_bdc_3_·nH_2_O			(Eu_1-x_Lu_x_)_2_bdc_3_·nH_2_O		
χ_La_ (at.%)	width, μm	length, μm	χ_Gd_ (at.%)	width, μm	length, μm	χ_Lu_ (at.%)	width, μm	length, μm
100	2.7 ± 1.2	8.7 ± 2.2	100	2.2 ± 1.3	5.8 ± 2.7	100	8.2 ± 3.6	12 ± 4
98	1.9 ± 0.6	5.3 ± 1.4	98	2.7 ± 1.1	5.8 ± 1.9	98	5.1 ± 1.5	8.0 ± 2.3
95	3.1 ± 0.9	8.5 ± 2.5	95	1.7 ± 0.6	4.2 ± 1.1	95	4.6 ± 1.2	7.5 ± 1.2
90	2.8 ± 1.0	7.4 ± 2.1	90	3.0 ± 0.8	7.4 ± 2.1	90	0.8 ± 0.3	4.9 ± 1.3
80	2.7 ± 1.0	6.5 ± 1.7	80	3.0 ± 1.0	7.0 ± 2.1	80	0.8 ± 0.4	3.5 ± 1.6
0	1.6 ± 0.8	3.9 ± 1.9	0	1.6 ± 0.8	3.9 ± 1.9	0	1.6 ± 0.8	3.9 ± 1.9

**Table 3:** Lifetimes (*τ*) and photoluminescence quantum yields (Φ_PL_) of (Eu_1-x_Ln_x_)_2_bdc_3_·nH_2_O (Ln = La, Gd, Lu; x = 0, 0.80, 0.90, 0.95, 0.98). The fractions of the exponential components are given in parentheses.

	(Eu_1-x_La_x_)_2_bdc_3_·nH_2_O		(Eu_1-x_Gd_x_)_2_bdc_3_·nH_2_O		(Eu_1-x_Lu_x_)_2_bdc_3_·nH_2_O		
χ_Ln_ (at.%)	*τ*, ms	Φ_PL_, %	*τ*, ms	Φ_PL_, %	*τ*_1_, ms	*τ*_2_, ms	Φ_PL_, %
98	0.45 ± 0.01	9 ± 1	0.47 ± 0.01	10 ± 1	0.41 ± 0.01 (91.4%)	1.54 ± 0.18 (8.6%)	16 ± 1
95	0.44 ± 0.01	10 ± 1	0.46 ± 0.01	12 ± 1	0.34 ± 0.01 (85.9%)	1.62 ± 0.08 (14.1%)	23 ± 1
90	0.45 ± 0.01	9 ± 1	0.46 ± 0.01	13 ± 1	0.46 ± 0.01	-	11 ± 1
80	0.44 ± 0.01	10 ± 1	0.46 ± 0.01	12 ± 1	0.46 ± 0.01	-	12 ± 1
0	0.43 ± 0.01	10 ± 1	0.43 ± 0.01	10 ± 1	0.43 ± 0.01	-	10 ± 1

**Table 4:** Asymmetry ratio R_as_ of (Eu_1-x_Ln_x_)_2_bdc_3_·nH_2_O (Ln = La, Gd, Lu; x = 0, 0.80, 0.90, 0.95, 0.98).

χ_La_ (at.%)	(Eu_1-x_La_x_)_2_bdc_3_·nH_2_O	(Eu_1-x_Gd_x_)_2_bdc_3_·nH_2_O	(Eu_1-x_Lu_x_)_2_bdc_3_·nH_2_O
98	3.8 ± 0.3	3.8 ± 0.3	3.0 ± 0.3
95	3.9 ± 0.3	3.8 ± 0.3	2.9 ± 0.3
90	3.9 ± 0.3	3.9 ± 0.3	3.5 ± 0.3
80	3.9 ± 0.3	3.7 ± 0.3	3.7 ± 0.3
0	3.7 ± 0.3	3.7 ± 0.3	3.7 ± 0.3

**Table 5:** The concentrations of REE ions in initial solutions used for the synthesis of (Eu_1-x_Ln_x_)_2_bdc_3_·nH_2_O MOFs.

χ_Ln_ (at.%)	C(Eu^3+^), mM	C(Ln^3+^), mM
100	0	5.0
98	0.1	4.9
95	0.25	4.75
90	0.5	4.5
80	1.0	4.0
0	5.0	0

**Table 6:** Eu^3+^ relative atomic fraction to the total amount of Eu^3+^ and Ln^3+^ (Ln = Lu, La, Gd) in (Eu_1-x_Ln_x_)_2_bdc_3_·nH_2_O compounds (x = 0, 0.80, 0.90, 0.95, 0.98, 1) taken during synthesis and obtained from EDX data.

(Eu_1-x_La_x_)_2_bdc_3_·nH_2_O		(Eu_1-x_Gd_x_)_2_bdc_3_·nH_2_O		(Eu_1-x_Lu_x_)_2_bdc_3_·nH_2_O	
χ_La_ (at.%), Taken	χ_La_ (at.%), EDX	χ_Gd_ (at.%), Taken	χ_Gd_ (at.%), EDX	χ_Lu_ (at.%), Taken	χ_Lu_ (at.%), EDX
100	100	100	100	100	100
98	97.4 ± 0.9	98	98 ± 1	98	98 ± 1
95	94.7 ± 0.6	95	95.4 ± 0.6	95	94 ± 3
90	89.4 ± 0.5	90	90.2 ± 0.8	90	91 ± 4
80	79.7 ± 2.3	80	81.1 ± 1.2	80	79 ± 6
0	0	0	0	0	0

## Data Availability

The data presented in this study are available in the article.
